# An online discussion between students and teachers: a way forward for meaningful teacher feedback?

**DOI:** 10.1186/s12909-021-02730-8

**Published:** 2021-05-21

**Authors:** Agra Dilshani Hunukumbure, Philippa Jane Horner, Jonathan Fox, Viral Thakerar

**Affiliations:** 1 Hillingdon Hospital NHS Foundation Trust, Pield Heath Road, Uxbridge, UB8 3NN UK; 2 Imperial College London, Exhibition Road, South Kensington, London, SW7 2BX UK

**Keywords:** Teacher-evaluation, Online feedback, Open discussion, Challenges, Two-way feedback, Undergraduate, Barriers

## Abstract

**Background:**

Student evaluation is an essential component in feedback processes in faculty and learner development. Ease of use and low cost have made paper evaluation forms a popular method within teaching programmes, but they are often seen as a formality, offering variable value towards the improvement of teaching. Students report poor motivation to engage with existing feedback tools whilst teachers describe receiving vague, contradicting, or irrelevant information.

We believe that feedback for teachers needs to be a two-way process, similar to feedback for students, for it to be effective. An online feedback tool has been implemented for third-year medical students from Imperial College London to promote open discussion between teachers and students. The feedback tool is accessible throughout students clinical attachment with the option of maintaining anonymity. We aim to explore the benefits and challenges of this online feedback tool and assess its value as a method for teacher feedback.

**Methods:**

Qualitative data was obtained from both volunteer third-year medical students of Imperial College London and Clinical Teaching Fellows using three focus groups and a questionnaire. Data was analysed through iterative coding and thematic analysis to provide over-arching analytical themes.

**Results:**

Twenty-nine students trialled this feedback tool with 17 responding to the evaluative questionnaire. Four over-arching themes were identified: reasons for poor participation with traditional feedback tools; student motivators to engage with open feedback; evaluative benefits from open feedback; concerns and barriers with open feedback.

**Conclusion:**

This feedback tool provides a platform for two-way feedback by encouraging open, transparent discussion between teachers and learners. It gives a unique insight into both teachers and peers perspectives. Students engage better when their responses are acknowledged by the teachers. We elaborate on the benefits and challenges of public open feedback and approaches to consider in addressing the self-censorship of critical comments.

**Supplementary Information:**

The online version contains supplementary material available at 10.1186/s12909-021-02730-8.

## Background

Meaningful feedback is essential for both faculty development and programme evaluation [1]. Inclusion of the student voice within evaluation and development processes can empower students [2], improve academic performance [3], increase learner-satisfaction [2], improve quality of teaching [4] and assist in the professional development of educators [5].

Student feedback gathered through paper evaluation questionnaires completed at the end of a teaching course is a common and easy way of obtaining feedback from large groups [6,9]. However, for many it has become a formality [1]. Furthermore, there is ongoing debate about the reliability and reproducibility of student feedback [1, 10, 11]. Students have previously reported feeling rushed to complete paper feedback forms promptly, further compromising the value of comments available to teachers [12].

Anecdotal evidence from working with clinical teaching fellows (CTFs) affiliated with Imperial College London shows that they receive little effective feedback from their teaching evaluations. An online anonymous survey of CTFs has identified non-specific feedback, contradicting [student] opinions and complaints about issues beyond CTF control as significant limitations. Comments from the students suggest that their feedback is never acted upon. Several studies resonate with this: Aslam demonstrates that there is a considerable disagreement among the students responses [1]; Svinicki argues that through learned helplessness students find little reason to submit feedback which they believe will not affect future teaching [13]; Day reports questionnaire fatigue as another reason for poor engagement [6].

In order to optimise the evaluation process, students and teachers must use a means of gathering information that is acceptable to all parties [1]. When constructed well, online tools offer a simple, cost-effective method of collecting feedback from large groups of learners [9]. A study by Shabbir et al. demonstrated a significant increase in the response rate with use of online form compared a paper evaluation form [14]. Online feedback is not a new approach but, to ensure evaluations are valid, responses must be collected at appropriate times, requested throughout teaching programmes, and returned with an adequate response rate [9, 11]. Some teachers at Imperial College London have used Mentimeter (an online software in which the audience can respond to questions using smartphones) to evaluate the teaching sessions. The students responded better as there was no further forms or links [15].

As a possible answer to some of the common problems when evaluating teaching, we have created an online feedback tool. Though this tool could be used to evaluate any teaching session, it is designed for use by a group of students who have lengthy clinical placements (4 weeks or more) and have regular sessions with a group of teachers.

### Context

We piloted the online feedback tool with third-year medical students from Imperial College London attached to Hillingdon Hospital for a ten-week clinical placement. These students had regular teaching sessions on clinical skills, bedside teaching, and lecture-based teaching with local CTFs. CTFs are junior doctors at an early stage of their teaching careers who are keen to develop their teaching skills. They usually have one-year teaching contracts outside of their postgraduate training programmes. In current practice, students give formal feedback to teachers by two different methods: paper evaluation forms given out by the individual teachers after their teaching sessions and the college central feedback tool, Student Online Evaluation (SOLE). SOLE is an online questionnaire circulated by email during the last week of each placement.

### The novel online feedback tool

Our vision is to promote dialogue between students and teachers. We believe that feedback for teachers should be a two-way process, as is the case when learners receive feedback [16, 17]. Discussions may lead to a better mutual understanding between teachers and students. This encourages deeper, reflective learning for students and enhanced development for teachers [18]. However, the authority and power within a student-teacher relationship often lie with the teacher, thus the same approaches of providing feedback to students may not be practically applicable to teacher feedback [19]. We believe that this online feedback platform may help to ameliorate the impact of this power imbalance.

**Fig. 1:**
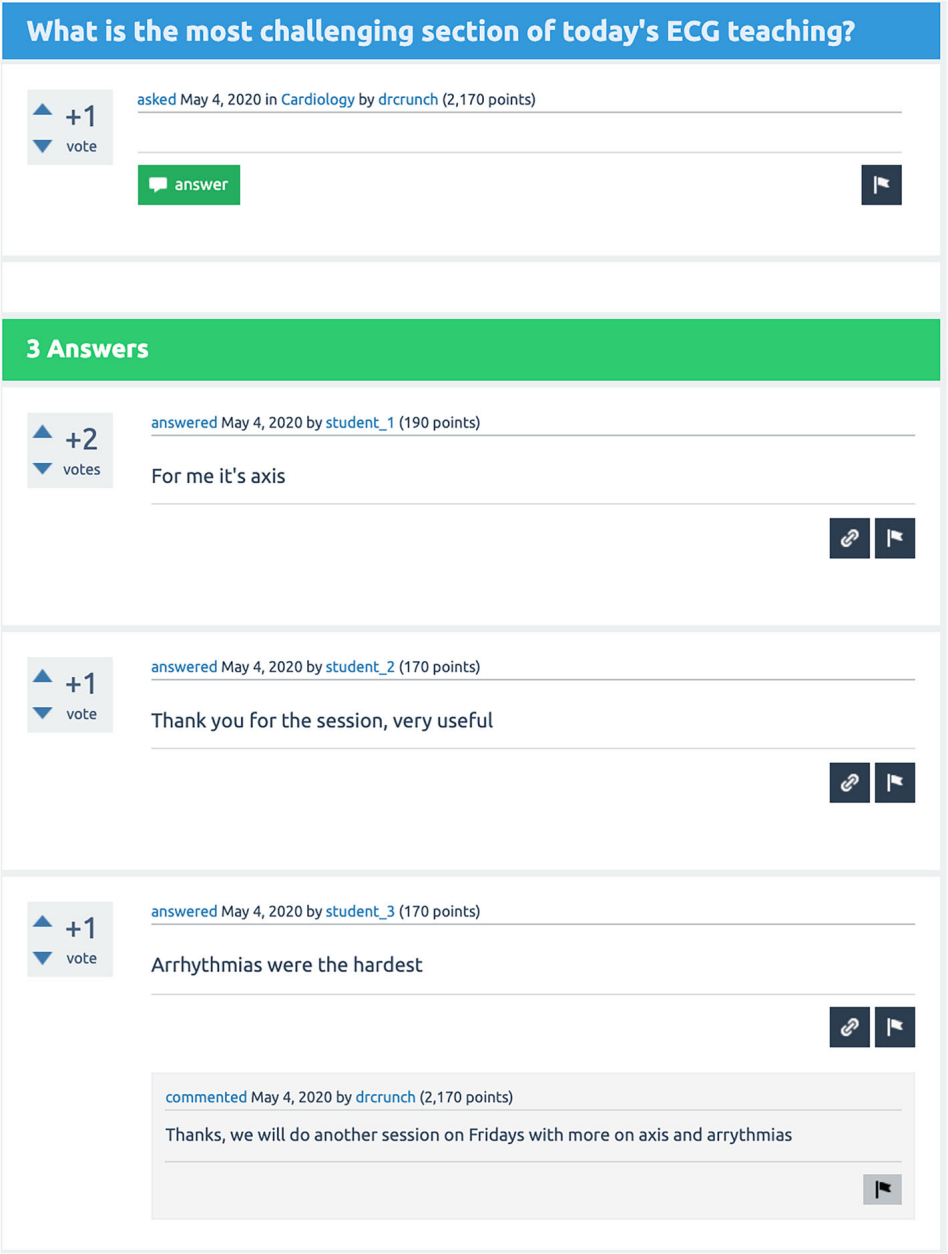
Screenshot from a reconstruction of an online discussion between a teacher and the students. Students may choose to use a pseudonym or remain anonymous. The comments above are not by real students but are representative of a typical discussion

The students are given access to the platform at the induction of their placement. The platform is based on the open-source PHP/MySQL platform Question2Answer (https://www.question2answer.org/).Throughthis online platform they can post feedback about teaching, ask CTFs questions and make requests regarding future teaching. They can view and annotate each others comments and may also agree or disagree with statements by *up-voting* or *down-voting* respectively. CTFs can address the feedback statements with relevant explanations or actions. Students and CTFs can access the tool throughout the placement and students have the option of remaining anonymous when using it. An example of such an online discussion is shown in Fig.1.

Teachers can seek feedback on many educational aspects: the teacher, the content of a teaching session or a whole programme [11]. Control of this tool lies with teachers. When teachers use this tool, they have the option to choose the focus of their feedback by tailoring questions or statements posed to the students. These could include the topic of a future session, a question to evaluate their understanding or a question about a specific aspect of a previous teaching session.

The online conversations between students and teachers or among peers are visible to all the users. We have referred to users comments as open or public to indicate this visibility throughout this paper.

### Aims

In this study, we explore the students and teachers perspectives on the benefits and challenges of this approach and assess the tools perceived value to the teachers.

## Methods

For this qualitative study, we have adopted a social constructionist view and have embraced a neutral stance to data collection and analysis to explore both the benefits and the challenges of this feedback approach. Balanced analysis facilitates further development of this tool and informs those interested in adopting similar approaches in their own practices.

### Study participants and recruitment

The study participants were volunteer third-year medical students from Imperial College London, undertaking a ten-week clinical placement at Hillingdon Hospital from March to May 2018 and clinical teaching fellows who provided regular teaching sessions and used this tool during this attachment. All twenty-nine students on the placement were given access to the online feedback tool from the start of the placement, and engagement with the tool was voluntary. Participants for the focus groups were recruited from amongst these twenty-nine students through advertisements in the student common room and through their undergraduate teaching coordinator. They were rewarded with a certificate of appreciation for their contribution. Ten students (six females, four males) took part in two focus groups which were conducted at the end of the placement. Questionnaires were given out to all students to evaluate the overall view of the cohort and seventeen out of twenty-nine eligible students returned the completed questionnaire.

### Data collection

Data were collected from April to May 2018 using questionnaires comprising both closed and open questions, and three focus groups, two with five students each and a third with three teaching fellows. Pre-determined questions were used to guide the discussion resulting in the generation of rich ideas (please see appendix 1 and 2 for the question guides used for students and teachers respectively). The lead researcher acted as the moderator. Discussions were audio-recorded and later transcribed in verbatim by the researchers. All third-year students were given a paper questionnaire with an envelope and were asked to return the filled forms to the teaching coordinator.

### Data analysis

Two of the researchers were clinical teaching fellows involved in teaching the participant medical students. The dual role of being a teacher and a researcher can lead to a greater understanding of themes and contextualisation of the data [20]. However, this presented a potential risk of unconscious bias towards magnifying the benefits of the online feedback tool. Nevertheless, having adopted a neutral stance the researchers were equally interested in exploring the challenges and believed in a balanced assessment to establish the effectiveness of this platform. The authors who performed the analysis were not involved in teaching the study participants.

The data was analysed using the framework for thematic analysis as described by Braun & Clarke [21]. Three members of the research team familiarised themselves with the data before generating codes through inductive, open coding processes. With constant comparison and discussion amongst the authors, axial coding ensured codes were assessed for similarities, differences, and duplicates. Two of the authors performed further analysis to generate descriptive themes and categorise associated codes. Each descriptive theme was evaluated with the corresponding data to ensure they correctly reflected the original data. Finally, descriptive themes were assessed to identify over-arching analytical themes. The data from the questionnaire did not add additional themes to those from the focus groups.

Throughout this process, an iterative approach of critical evaluation was encouraged with both individual and group reflections to identify conflicts and discrepancies in codes or themes. Any differences were resolved through discussion and further analysis of data when needed. Prior to advancing from each stage, summaries were distributed amongst the team to ensure that all agreed.

### Ethical approval

Ethical approval was obtained from the Medical Education Ethics Committee of Imperial College London (MEEC171887).

## Results

All students created an account for the online feedback tool at their induction, with the option to use a pseudonym if they wished. The response rates were particularly high when CTFs posed a specific question or invited a consensus.

Four overarching themes emerged from the analysis of our data; from focus groups and questionnaires: 1) reasons for poor participation with traditional feedback tools; 2) student motivators to engage with open feedback; 3) evaluative benefits from open feedback; 4) concerns and barriers to open feedback. These themes, along with a summary of the associated descriptive narratives and illustrative quotes are shown in Tables 1, 2, 3 and 4. In illustrative quotes, a and b represent the two student focus groups, M and F denote males and females, respectively. Each male and female student was also given a number to distinguish between focus group members. CTFs were anonymised with a number. Quotes from student questionnaires are identified by SQ.

### Reasons for poor participation with traditional feedback tools (please refer to Table1)

Multiple reasons were identified for poor engagement with paper-based questionnaires and the student online evaluation (SOLE). These included the delay between the experience and giving feedback, the inconvenience of completing the feedback, a lack of purpose in providing feedback and feeling neutral towards the teaching.

**Table 1:** Illustrative quotes and associated reasons for poor participation with traditional feedback tools

Theme	Narrative example	Illustrative Quotes
Reasons for poor participation with traditional feedback tools	Delay between teaching and the opportunity to give feedback	bF1: I cant even remember the lecturer when Im in the thing, let alone 3 months after.
	More convenient and less time-consuming to give thoughtless feedback	aM2: I find [with] the paper feedback, you usually tick all the boxes so you can leave early. bF1: The forms are the longest thingsit takes 5min to read the first pageand then 5min to read each subsequent page.
	No expectations on seeing changes that directly benefits the giver	bM1: We dont like doing things we dont gain anything from. not going to benefit you directly. aM1: Theres no way for us in medical school to directly give feedback that can be changed in the same [academic] year.
	Not having an extreme opinion about a teaching experience	aF1: Ive only used SOLE when its something really negative

### Student motivators to engage with open feedback (please refer to Table2)

Student participants described that open feedback allowed teachers to acknowledge and respond to them and establish a dialogue, as well as provide transparency and accountability from faculty. Students also appreciated gaining an insight into faculty thought processes. The immediacy of the teachers response generated a perception of personal benefit among students, leading to greater motivation to participate.

**Table 2:** Illustrative quotes and associated motivating factors to engage with open feedback

Theme	Narrative example	Illustrative Quotes
Motivators to engage with open feedback	Acknowledgement from teachers in response to feedback given; someone is accountable for responding to student feedback	aM3: Regularly updating the website and just answering feedback questions, just to make people know that we [teachers] have looked at it. to reinforce, to give them that reassurance that things are being done.
	Transparency of the evaluation process	bM2: You write your feedback and you know exactly who it is going to with other feedback systems you are writing it but you dont know who you interacting with and [who is] reading it. bF1: You can see what the faculty is thinking
	Higher likelihood of students directly benefitting from the feedback they give	aM2: If [students] get engaged early on they [CTFs] can then see the feedback, change, adapt to it. aM1: Ifeveryone is confused about something in gastro, then that weeks [teaching is focused on] upper limb we could deliver some gastro there as well and make it [the teaching] focused more on what the group want.
	Having dedicated time to provide feedback	aM2: I dont think anybody wouldjust fill out feedbackspontaneouslythere has to be a time and a place. CTF1: They all engage when we are in a session and then dedicate the last 5 minutes to giving feedback.
	Seeing evidence of other students participation	aM1: Im more likely to put a response in if I know that other people have written stuff as well.

### Evaluative benefits from open feedback (please refer to Table3)

The faculty appreciated the opportunity to clarify feedback from students and gained further information regarding aspects of teaching. All participants agreed that giving students insight into their teachers perspectives would likely lead to greater mutual understanding and foster mutual respect. Participants indicated greater satisfaction with the process when teachers used this information to inform future practice within the timeframe of their clinical placement. Additionally, teachers recognised the opportunity to learn from their peers through comparison and subsequent self-appraisal.

**Table 3:** Illustrative quotes and associated evaluative benefits from open feedback

Theme	Narrative example	Illustrative Quotes
Evaluative benefits from open feedback	Learning from the feedback given to colleagues	CTF2: Seeing how other people things do is so critical to everyones development. CTF1: [If] one particular teacher was getting lots and lots of positive feedback that would make me more interested in what was different.
	Opportunity to respond to feedback and correct misunderstanding	CTF2: If something is been misunderstood you have the opportunity to reply and correct miscommunication in a respectful way taking on-board the concerns, saying what you think may give more colour or context to it or what should be done differently.
	Ability to get more information from students	CTF1: It actually makes it a two-way process then rather than just a one-way process of getting a paper feedback form. bM2: I think the idea of seeing what someone else has said you might not necessarily think of something, when someone else has put it you can agree with it allows everyone to get a better input
	Evaluation of group consensus prioritisation of key issues	bM1: The up-vote [is] useful it shows everyone is thinking about what you are thinking. Also makes it easier for changes to be made for something like paper feedback you cant really put it all together and say that each and every person is saying the same thing. But with this you can see that there is some sort of agreement and its a lot easier and quicker which is of benefit. SQ14: Vote up is useful for supporting feedback
	Student insight into peer & teacher perspectives	aM2: its quite useful because you see other peoples views, it opens your views as well, so you see things from different perspectives. bF1: But sometimes seeing what everyone else has written makes you realise that you probably have missed something or that you have not thought about it. CTF2: Students would see each others feedback which was a new window to explore for them. They dont often get that opportunity. We could actually get consensus on certain things
	Responsibility to create meaningful feedback	CTF2: the quality of the free text answers they gave were sometimes really good perhaps influenced by the fact that other people might be looking at them

### Concerns and barriers to open feedback (please refer to Table4)

The researchers found no critical comments or down- votes on the online feedback platform, thus we explored the underlying reasons during focus groups. Many students felt uncomfortable submitting critical feedback, particularly in an open forum. A CTF postulated this might be due to the established rapport between students and teachers over a relatively long period of time. Some students felt deterred from submitting their feedback if their opinion contradicted the consensus. Both student and CTF participants agreed that these could be overcome using anonymous feedback and options to deliver closed feedback directly to an individual.Though there was no critical feedback, teachers were concerned that this would be visible to colleagues and students. CTFs acknowledged that any critical feedback could have an emotional impact and an open forum could amplify this.

**Table 4:** Illustrative quotes with associated concerns and barriers to open-forum online feedback

Theme	Narrative example	Illustrative Quotes
Concerns and barriers with open-forum online feedback	Group consensus deterring students from offering personal or differing opinions	aM3: I guess if you see someone put something down that you were going to put but the opposite, it does put you off a bit, knowing it is anonymous might make you feel better if you still feel comfortable you can put it down
	Student teacher relationship reducing critical feedback	CTF1: they want to maintain anonymity they feel bad [because] we build personal rapport with the students feel bad giving really critical feedback to someone or on a session if it was in the public domain
	Critical feedback visible to colleagues and faculty	CTF3: Im thinking if someone gave me really bad feedback in an open way all the students would see, all the teachers would see it, I feel I need to defend my position thats something I worry about.
	Critical feedback visible to other students	CTF1: I wonder if the public domain does make the person less likely to be critical maybe having that hidden element might be something to encourage people to give critical feedback bM1: there should be an option to make it closed you should be able to put a tick whether you want it to be seen to the public or not
	Larger emotional impact if critical feedback in a public forum	CTF1: I can think of people who may not respond as openly and constructively with public criticism. This is why I think it is very much dependent on the personality of the teacher. CTF2: If someone gives you negative feedback for a real reason, there will always be an emotional element that emotional element could be amplified by it [being] accessible compared to just privately to you that risk is being weighed up against the overall benefits of the transparency of the system
	Access to the platform	SQ12: An app would be more easy to use and access and would not require repeated logins an app would encourage use bF2: The most important thing for any feedback for it to be easy to access so instead of typing it having app or ready link. CTF2: A brief customised app might be easier because everyones got an iPad at Imperial, or just make it compulsory in the first session to [load it] onto your home screen for a one touch solution to give feedback.

## Discussion

Our feedback tool offers a novel approach for students and teachers to engage in a dialogue about learning and teaching, going beyond traditional feedback methods.

### Challenges of the existing feedback approach

Our observations of shortcomings in existing feedback approaches aligned with those presented in prior literature: delayed requests from teachers for feedback, lengthy feedback forms, no personal benefits perceived by students, student-determined necessity of feedback as perceived really negative or really positive teaching experiences, ease of offering undiscerning feedback rather than constructive comments, and perceived pressure to respond positively in the presence of a teacher [1, 7, 12, 13]. In the following sections, we have investigated our feedback tool in view of these challenges.

### Unique insight into others views

The public nature of this approach provides an invaluable insight into the perspectives of teachers, students and of their respective peers. The comments and their weighting (through up-votes; no down-votes received) establish a group opinion, which is more difficult to elicit from individual feedback. Platform users learn about the conflicting requests of students, challenges for teachers, limitations of resources and most importantly, the reasons why some of their feedback is not acted upon. Therefore, student and teacher opinions in an open platform accessible to all are invaluable. Highlighting this benefit, one student participant shared an experience as a member of the student union where unnecessary steps were taken following a request from a demanding student, unaware of the views of the majority. This is further affirmed by a letter published by the students from the same institution highlighting that SOLE feedback is biased as most responses are from students with negative experiences [15]. Though uncomfortable for the student concerned, allowing them to see others viewpoints may improve their awareness of the group consensus and gain a better understanding of the wider circumstances.

The study of Robins et al. demonstrates that teachers are more likely accept feedback from students who are engaging and performing academically well [19]. Open group consensus may provide a holistic picture and also guides teachers in choosing future teaching topics, facilitating other teaching improvements, and enhancing student engagement.

### Motivation for participation

Teachers acknowledgements of their responses and seeing peer contributions incentivises students ongoing participation in this dialogue. The transparency and accountability of the process offered a further motivation for students to engage as demonstrated by Dudek at el [18]. Therefore, students commitment is closely guided by the teachers enthusiasm and drive to respond appropriately and promptly.

We argue that a teachers mindset also needs adapting to this new approach, where the feedback process does not end with a teaching session, but the dialogue continues. Some teachers may not foresee the value of investing time responding to students or analysing their comments until they experience the benefits. Setting aside a few minutes each day is helpful. This tool is accessible by all the relevant teaching staff, therefore the demand on a single teacher is less.

Despite earlier literature raising concerns about the accessibility of online systems [12], our experience suggests that online access is no longer an issue in the current digital era where almost all students have access to smartphones or hand-held computers. *Unrestricted* access to the tool throughout their placement may also have contributed to the better engagement.

Nonetheless, both students and CTF participants believed that easy access to the platform is a key success feature and further development of the tool into a phone or tablet application with one-time log-in would likely enhance engagement.

### Public feedback

Despite no critical comments being received, such comments being visible in the public domain was a concern to the CTFs. One could argue that any critical comments can cause an emotional stir, regardless of being private or public. In a private setting, teachers deal with it in isolation while with open feedback, the emotional impact may be heightened but the affected teacher may get support and guidance from their peers. Open feedback can also promote self-reflection amongst other teachers, share good teaching approaches and support planning for future development, similar to the insight that may be gained from participating in the peer review process [22, 23].

Lack of critical comments posted on the platform may suggest students were practising self-censorship and this is a concern for the researchers. Several students disliked making contradicting statements or critical comments on a public platform. Therefore, all agreed that a successful tool must have options to send sensitive feedback privately to an individual teacher, the teaching coordinator, or an appropriate person in the university.

In our social norms, we tend to focus our conversations on the positive aspects, especially in formal relationships [24]. It is well-known that some teachers avoid giving critical feedback to students for several reasons including damaging relationships [25, 26]. We believe that these reasons apply to students, perhaps to an even greater extent, as discussed under power imbalance below.

### Power imbalance between student and teacher

Whilst not elicited as a concern amongst the students participating in this study, we argue that there are several implicit barriers to providing critical feedback to teachers. Firstly, there are the socio-cultural norms of the student-teacher relationship and the perceived power imbalance, where the authority lies with teachers [22, 27]. Secondly, the student-teacher relationship is built over a period, which could favour positively skewed feedback or avoidance of making any critical comments. Thirdly, students could view feedback as a way of showing gratitude or appreciation for their teaching. Olvet et al. demonstrate that medical students are reluctant to provide constructive feedback to the teacher even when feedback is anonymous and has no influence on their future grades. In this study, students have displayed a culture of politeness, softer and passive language with qualifying negative with positive comments known as hedging [28]. Reluctance to provide critical feedback is therefore a universal challenge irrespective of the mode of feedback.

We make several suggestions to alleviate the issue. Explaining the purpose and benefits of constructive teaching evaluations and posting guided questions may encourage students. Highlighting the option to post anonymously and assure students that negative comment will have no impact on their future teaching or assessment is imperative. Providing guidance on how to formulate feedback to teachers may also empower students [14, 19].

### Feedback but not feedback: reframing feedback

We examined the use of this online platform and found that some students accessed it in their own time, away from teaching premises, to respond to certain teacher questions. Interestingly, during our focus group discussions, the students expressed a preference for having allocated time for giving feedback within a teaching session. This raised the possibility that the word feedback could unnecessarily formalise the process and hinder unbiased open discussion. By asking varied questions on the platform without labelling them as a means of gathering feedback, we argue that student discussion may subsequently increase. In this way, the teacher can direct the conversation to gain a better understanding of the students opinions. Rebranding the platform using a word without the historical connotations associated with feedback may be a productive way forward.

### Limitations

Our sample size was small and therefore may not be representative of the whole student cohort, thus we cannot generalise the findings. Nevertheless, our rich data gave an insight into the use of this tool in our context.

In this study, we focused on teachers receiving feedback through dialogue with students. The validity and reproducibility of the feedback from the students can be influenced by many factors [10]. It is beyond the scope of this study to establish these factors. Further research exploring the reflections of teachers who received both paper-based private feedback and this online approach would be useful to further explore the value of this tool.

As discussed previously, the teachers did not receive any negative feedback from the students. We explored the possible reasons for this behaviour and the suggestions to mitigate such an issue under Power imbalance between student and teacher. Our discussion is based on our own arguments and related literature.

## Conclusion

This online feedback platform has engaged both teachers and learners in a conversation providing insight into each others perspectives. Teachers could respond to students comments and have the flexibility to direct a focused dialogue and establish a consensus. Students get the unique opportunity to appreciate their peers views and contribute to the group discussion. Students self-censorship of negative comments could be a universal challenge to any feedback approach while multiple steps could be implemented to mitigate and minimise the effects of public critical feedback. We believe that online platforms encouraging open dialogue can be a way forward for the digital age of teacher feedback.

## Supplementary Information

**Additional file 1.** Question guides for focus groups of students and teachers.

**Additional file 2.** Questionnaire on the online feedback tool.

## Data Availability

All the relevant data are included in the manuscript; Tables 1, 2, 3 and 4.

## Abbreviations

CTF: Clinical Teaching Fellow
SOLE: Student Online Evaluation
