# Supplementary material for: An online discussion between students and teachers: a way forward for meaningful teacher feedback?
Source: BMC Med Educ. 2021 May 21;21:289. doi: 10.1186/s12909-021-02730-8 (PMC8139045; doi:10.1186/s12909-021-02730-8)
Supplement: Supplementary file 1 — Additional file 1. Question guides for focus groups of students and teachers. [file 12909_2021_2730_MOESM1_ESM.docx]

Questions for Focus Groups - Students

1. What types of teacher feedback tools have you used in the past?
2. Have you used this new online tool?
   1. What prompts you to give feedback with this tool?
3. How did you find the new tool?
   1. What were the benefits?
   2. What were the challenges?
4. How did you feel that your feedback would be visible to other students?
   1. Did you feel your feedback was influenced by it being visible to other students? In what way?
5. How did you feel about being able to see other students' feedback?
6. How did you feel about being able to vote up/down students' feedback?
   1. Did this influence your feedback?
7. Any thoughts for improvement / comments that we have not covered before

Questions for Focus Groups – Teacher

1. What types of teacher feedback tools have you used in the past?

How often do you collect feedback from students?

1. How did you find the new tool?
   1. What were the benefits?
   2. What were the challenges?
2. How did you feel that your feedback would be visible to fellow teachers and all students?

Prompts -Any negatives, any positives

1. How did you feel about being able to see other teachers' feedback?
2. How did you find the vote up/down component?
   1. Did this influence you in any way?
3. Any ideas for improvement /areas we should modify
4. Any comments / thoughts that we have not covered before
