# Supplementary material for: An online discussion between students and teachers: a way forward for meaningful teacher feedback?
Source: BMC Med Educ. 2021 May 21;21:289. doi: 10.1186/s12909-021-02730-8 (PMC8139045; doi:10.1186/s12909-021-02730-8)
Supplement: Supplementary file 2 — Additional file 2. Questionnaire on the online feedback tool. [file 12909_2021_2730_MOESM2_ESM.docx]

Questionnaire on the online feedback tool

1. Did you use the online feedback tool?

Yes No

If you did not use this tool: is there a reason?

……………………………………………………………………………………………………………………………………..

……………………………………………………………………………………………………………………………………..

1. How user-friendly was the tool?

(Not user-friendly at all) 1 2 3 4 5 (Extremely user-friendly)

1. How did you find this tool as a method of evaluating your teaching session?

(Not useful at all) 1 2 3 4 5 (Extremely useful)

Please explain the reasons. Do you have any positives or negatives comments?

……………………………………………………………………………………………………………………………………..

……………………………………………………………………………………………………………………………………..

1. How did you find this tool as a method of giving individual teacher feedback?

(Not useful at all) 1 2 3 4 5 (Extremely useful)

Please explain the reasons. Do you have any positives or negatives comments?

……………………………………………………………………………………………………………………………………..

……………………………………………………………………………………………………………………………………..

Please turn over

1. In your experience, when comparing with paper evaluation forms, how did you find this online tool?
   1. Positive comments: ………………………………………………………………………………………….

………………………………………………………………………………………………………………………….

- 1. Negative comments: ………………………………………………………………………………………..

………………………………………………………………………………………………………………………….

1. How do you feel about using the vote-up and vote-down function of this tool?

……………………………………………………………………………………………………………………………………..

……………………………………………………………………………………………………………………………………..

1. Do you have any suggestions for how this tool can be improved?

……………………………………………………………………………………………………………………………………..

……………………………………………………………………………………………………………………………………..

……………………………………………………………………………………………………………………………………..

……………………………………………………………………………………………………………………………………..

……………………………………………………………………………………………………………………………………..

**THANK YOU**
